# A novel method for the extraction of outer membrane vesicles (OMVs) from *Bordetella pertussis* Tohama strain

**Published:** 2020-02

**Authors:** Mohammad Sekhavati, Ashraf Mohabati Mobarez, Seyed Davar Siadat, Mojtaba Noofeli

**Affiliations:** 1 Department of Bacteriology, Faculty of Medical Sciences, Tarbiat Modares University, Tehran, Iran; 2 Microbiology Research Center (MRC) Pasteur, Institute of Iran, Tehran, Iran; 3 Department of Human Bacterial Vaccine, Razi Vaccine and Serum Research Institute, Agricultural Research, Education and Extension Organization (AREEO), Karaj, Iran

**Keywords:** *Bordetella pertussis*, Extraction, Outer membrane vesicle, Vaccine

## Abstract

**Background and Objectives:**

There are many pertussis outbreaks which is mainly due to the reduction in the immunity of acellular pertussis (aP) vaccines. Therefore, there is a crucial necessity to develop a new generation of pertussis vaccine. Preceding researches have shown that *Bordetella pertussis* outer membrane vesicles (OMVs) have appropriate specifications, making them a suitable vaccine candidate against pertussis.

**Materials and Methods:**

The OMVs were separated by a new serial ultra centrifugation technique. Transmission electron microscopy (TEM) examination, SDS-PAGE, Western blotting and ELISA assay were used to characterize the OMVs.

**Results:**

TEM studies showed the size of the extracted OMVs at 40–200 nm. The presence of pertussis toxin, filamentous hemagglutinin, and pertactin was verified using Western blot and ELISA assay.

**Conclusion:**

The presented technique is a simple and effective way to obtain OMVs from *Bordetella pertussis.* So it can be utilized as an appropriate procedure in the development of an OMV-based vaccine against pertussis.

## INTRODUCTION

Pertussis (whooping cough) or 100-day cough is a well-known contagious infection of the respiratory tract, especially in infants. It caused by the fastidious, Gram-negative bacterium *Bordetella pertussis* (1, 2). Notwithstanding numerous vaccination strategies, pertussis is still a prominent concern of the world and a life-threatening childhood disease, though it is preventable by vaccination (3–5). There are varying sources for pertussis, but family members are the main source of the disease, which is often spread through direct contact from person to person (6, 7). First-generation pertussis vaccines (killed vaccines) introduced in the 1950s. Currently, owing to its favorable efficacy, the killed pertussis vaccines are utilizing in many countries of the world. (8, 9). Based on a report from WHO, the infant’s death declined by around 100,000 cases from 1999 to 2014 after pertussis vaccinations (10). Owing to uncertainty about the safety of the whole-cell pertussis (wP) vaccines and its side effects, these vaccines were replaced with the safe acellular pertussis (aP) vaccines, comprising immunogenic antigens of *Bordetella pertussis (B. pertussis)* (11, 12). Although this switch between the two vaccines reduced vaccine side effects, it led to the outbreak and re-emergence of pertussis due to low efficacy and short-term immunity caused by aP vaccines (13–16). Meanwhile, aP vaccines containing alum, as an adjuvant, mainly induce Th2 (humoral) response while Th1/Th17 (cellular) response induced by wP. The former assisting in reducing protection, but the latter results in the induction of long-term immunity and clearance (2, 17).

Outer membrane vesicles (OMVs) are nanoparticles of spherical shape and a diameter of 10–300 nm. These nanospheres produced by Gram-negative bacteria and comprised of lipopolysaccharides (LPS), outer membrane proteins, and periplasmic proteins (18, 19). OMVs have recently suggested as one of the promising vaccine candidates for bacterial infections. The OMVs of *B. pertussis* composed of various major immunogens; therefore, they can be applied as a new and potent vaccines with low side effects (20–22). The current work presents a novel method for the extraction of OMVs from *B. pertussis*.

## MATERIALS AND METHODS

**Bacterial strain and animals.** *B. pertussis* Tohama strain and seven female BALB/c mice (4–6 weeks of age) for production of hyperimmune serum provided from Razi Vaccine & Serum Research Institute (RVSRI). All animal experiments were conducted in accordance with the procedures approved by RVSRI Animal Care and Use Committee (Karaj, Iran).

**Bacterial growth conditions.** A 20-μl aliquot of *B. pertussis* Tohama phase I strain was cultured on the Bordet-Gengou agar plate. A few small colonies were sub-cultured on Stainer-Scholte liquid medium for large-scale production of the cultures (23).

**extraction of OMVs.** The culture sample was incubated for ∼36 h (decelerating growth phase) and centrifuged at 7,000 × g at 4 °C for 45 min. The obtained pellet was washed twice with phosphate-buffered saline (PBS) and centrifuged at 10,000 × g for 15 min. The washed pellet was dissolved in a sodium chloride buffer (4 ml/g pellet) and homogenized by pipetting several times, to make a uniform suspension. After centrifugation at 10,000 × g for 15 min, the obtained pellet was treated with 0.1 M Tris-HCl, 10 mM EDTA pH 8 (6 times weight of the pellet) and homogenized by shaking. Subsequently, the suspension was sonicated in cool water for 5 min and then treated with 0.1 M Tris-HCl, 10 mM EDTA pH 7.5, sodium deoxycholate (5% W/V). 300 microliter of this Tris+EDTA+ sodium deoxycholate was added to 5 ml of sonicated suspension and mixed well. After 10 min the suspension was centrifuged at 10,000 ×g for 15 min. The supernatant was collected in a new tube and treated with 200 μl of 0.1 M Tris-HCl, 10 mM EDTA pH 7.5, sodium deoxycholate (5% W/V) and incubated for 10 min at room temperature. The treated supernatant was pelleted by centrifugation at 50,000 × g for 2 h at 4 °C. The pellet containing OMVs dissolved in 2 ml of sucrose 3% to make a suspension and then filtered through a 0.2 μm filter (Millipore, Germany). Finally, filter passing fluid that is containing of OMVs stored at 4 °C.

**Characterization of the extracted OMVs.** To evaluate the characterization of the extracted OMVs and comparing to previous studies several experiments were investigated as follows:

**Transmission electron microscopy (TeM) study of the extracted OMVs.** The extracted OMVs were suspended in ammonium acetate 0.1 M pH 7. Then 5 μl of the sample was dropped-cast onto a copper-coated grid. After staining with phosphotungstic acid, the stained grid, evaluated by using a transmission electron microscope (Zeiss EM10c, Germany).

**Protein assay.** Total protein concentration in OMVs was determined by the method of Bradford with bovine serum albumin as the standard (24).

**SDS-PAGE and Western blot analysis.** At first, 6 μg of the resultant OMVs was suspended in Laemmli sample buffer and run on SDS gels (12.5%) (25). Then the Western blot was used to confirm the main immunogenic proteins (26, 27). Briefly, after incubation of polyvinylidene difluoride membrane in 3% skim milk in PBS for 24 h, the SDS-PAGE protein bands were transferred onto it and washed three times with the washing buffer (PBS and 0.05% Tween 20) and then reacted with the commercial monoclonal antibodies such as anti-pertussis toxin (PTX), anti-pertactin (PRN), and anti-filamentous hemagglutinin (FHA). After three times of washing, the membrane treated with the anti-mouse IgG conjugated with alkaline phosphatase (at a dilution of 1:1000). After washing (three times), the result was visualized by adding 3,3`-Diaminobenzidines (DAB) substrate (Sigma, USA) to the membrane.

**Production of hyperimmune serum.** Hyperimmune serum against the extracted OMVs using complete/incomplete Freund’s adjuvant was prepared by three subcutaneous injections into five BALB/c mice as described previously (28). In detail, after homogenization 10 μl of the OMVs and 50 μl of complete Freund’s adjuvant per dose, it was injected subcutaneously into BALB/c mice at day zero. After booster immunizations (2 and 4 weeks after day zero), serum samples were gathered as hyperimmune serum after heart bleeding (day 14 after the second booster).

**ELISA assay.** The presence of specific antigens (PT, FHA, and 69-kDa) in the extracted OMVs examined by direct ELISA, by using standard antigens (NIBSC, UK) and OMV-hyperimmune serum as antibody. A checkerboard titration was developed to measure the optimum antigen concentration, which, herein, was considered as 120 ng/well for PT and FHA and 30 ng/well for 69-kDa protein. All the antigens were coated on a 96-well ELISA plate (eight wells for each antigen) and incubated at 4 °C overnight. After washing with PBS, the plate was blocked with the skim milk 3% (150 μl/well; Sigma, Germany) and incubated for 24 h at 4 °C. Subsequently, the plate was washed thoroughly, and an equal volume (50 μl) of each hyperimmune serum dilution (1:50, 1:100, and 1:200 in 3% skim milk) and also the normal mouse serum (NMS) (NMS; 1:50 in 3% skim milk) were admixed. Each serum dilution examined in duplicate for each antigen. The resultant mixture was finally incubated for 2 h at 37 °C. A number of wells were coated with 10 μg/ml of BSA and considered as the negative control. Following four-time washing of the wells, horseradish peroxidase-conjugated anti-mouse IgG (1:40,000; Sigma USA) was added at 50 μL/well, and the samples were incubated for 2 h at 37 °C. The wells were again rinsed four times to remove unbound antibodies. The plate was incubated with 100 μl of 3, 3′, 5, 5′-Tetramethylbenzidine in the dark for 10 min at room temperature. By the addition of 25 μl of the stop solution (HCl 5.8%), the reaction was stopped, and the absorbance was determined at 450 nm by the use of an ELISA plate reader (Stat Fax, USA).

## RESULTS

**Fig. 1.**
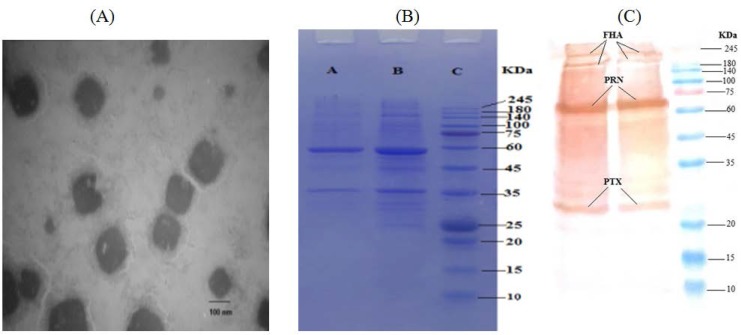
Characterization of the extracted OMVs. Panel A, electron microscopy image of negatively stained OMVs. Panel B, protein profiles of SDS-PAGE 12.5% (w/v); lanes A & B, the protein profile of the OMVs (in duplicate), lanes C, protein molecular weight marker. Panel C, Western blot of the OMVs using monoclonal antibodies (anti-PTX, anti-FHA, anti-PRN) in duplicate.

**Electron microscopy, protein content, and immunoblots.** For the assessment of the morphology of OMVs, the obtained sample was negatively stained and examined with a TEM (Fig. 1A). Nano-spherical shapes of 40–200 nm in diameter were seen similar to those reported previously (21). The protein content of the obtained OMVs was observed to be 220 μg/ml (24). In addition, the protein profiles of the OMVs were examined by SDS-PAGE (Fig. 1B), which verified the presence of famous immunogens such as PTX, FHA and PRN in the extracted OMVs (Fig. 1C).

**Fig. 2.**
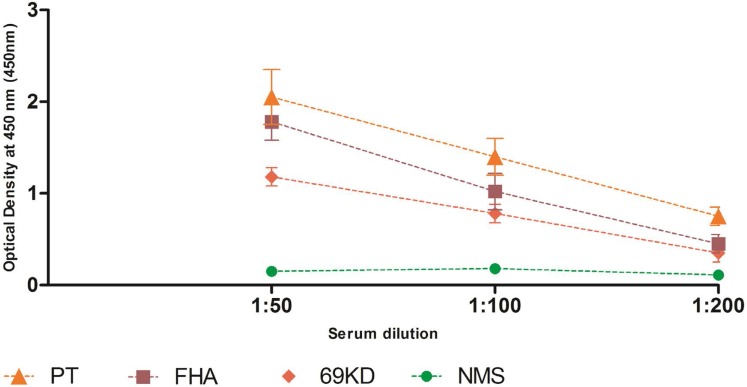
Antibody levels in the hyperimmune serum and NMS against PT, FHA and 69-kDa.

**The result of ELISA assay.** The evaluation of serological experiments confirmed that the obtained OMVs possess main immunogens such as PT, FHA, and 69-kDa (Fig. 2). As illustrated in this Figure, there was not any rise in the sera from the NMS, which was also affirmed by ELISA. Based on the data from Table 1, the absorbance value increased only in the pooled sample in all three dilutions; however, no elevation was observed in the NMS.

**Table. 1.** Optical density values of the hyperimmune serum and NMS (as antibodies) and PT, FHA and 69-kDa (as antigens) in ELISA assay.

**Sample name**	**Serum dilution**	**PT**		**FHA**		**69-kDa**	
		**A^450^ (plate 1)**	**A^450^ (plate 2)**	**A^450^ (plate 1)**	**A^450^ (plate 2)**	**A^450^ (plate 1)**	**A^450^ (plate 2)**
Hyperimmune	1:50	2.032	2.123	1.812	1.901	1.225	1.301
serum	1:100	1.520	1.495	1.011	0.982	0.841	0.798
Normal mouse	1:200	0.802	0.712	0.502	0.482	0.402	0.391
serum	1:50	0.156	0.132	0.135	0.165	0.145	0.136

## DISCUSSION

By virtue of Pertussis resurgence and the low efficient commercial aP vaccines, a more effective vaccine is highly needed to control the disease. *B. pertussis* OMVs are able to induce antibodies that obstruct the attachment of bacterium to the lung, thereby leading to no colonization (15). With having immune stimulators such as PTX, FHA, PRN, and LPS as adjuvants, these vesicles could perfectly provoke immune responses. Previous investigations have introduced OMV-based vaccines as a potent and reliable acellular vaccine (27, 29). In the process of OMV-based vaccine production, the type of extraction method is the most important step; therefore, using a simple and high efficient OMV extraction strategy will undoubtedly be helpful. Currently, sequential ultracentrifugation above 100,000 × g has been used for the extraction of pertussis OMVs. Unfortunately, this is not an easily-available technology in the majority of countries and most typically in the developing world. Consequently, a substitute procedure without very high-speed centrifugation is necessary.

The current study introduces a new method for the extraction of OMV from *B. pertussis*. The proposed strategy benefits from low-speed ultracentrifugation and good yield extraction of OMVs from *B. pertussis* Tohama strain, using different centrifugations maximum at 50,000 × g.

TEM analysis indicated that the extracted nano-sized spherical vesicles from Tohama strain in the range from 40 to 200 nm. The results of SDS-PAGE also showed several immunogenic proteins, including FHA, PTX and PRN, which affirmed by both Western blot and ELISA assay. Meanwhile, the findings attained by our method on the characterization of the extracted OMVs were consistent with those of previous studies utilized high-speed ultracentrifugation (26, 27). At least 10 independent repetitions of OMVs extraction and characterization processes were conducted with the proposed method. Interestingly, all the OMVs displayed similarity in morphology, size and the presence of main immunogens. Our findings suggest that the presented method is an efficient technique for extracting OMVs from *B. pertussis* and could be appropriate for the production of pertussis OMV-based vaccines, but immunogenicity must be check by valid potency test and field trials in separate researches.
